# London Lancet

**Published:** 1865-05

**Authors:** 


LONDON LANCET.---The April number of this old representative of English periodical medical literature, is promptly on our table, and filled with the usual variety of interesting and important articles.

